# Atlas of cortical sulcal imprints on *Pan* endocasts

**DOI:** 10.1111/joa.70185

**Published:** 2026-06-07

**Authors:** Edwin J. de Jager, Emmanuel Gilissen, Laurent Risser, Céline Amiez, Caroline Fonta, Muriel Mescam, Amélie Beaudet

**Affiliations:** ^1^ Department of Archaeology University of Cambridge Cambridge UK; ^2^ Laboratoire de Paléontologie, Évolution, Paléoécosystèmes et Paléoprimatologie (PALEVOPRIM) UMR 7262 CNRS & Université de Poitiers Poitiers France; ^3^ Department of Anatomy, School of Medicine, Faculty of Health Sciences University of Pretoria Pretoria South Africa; ^4^ Department of African Zoology Royal Museum for Central Africa Tervuren Belgium; ^5^ ULB Center for Diabetes Research Université Libre de Bruxelles Brussels Belgium; ^6^ Univ Toulouse, CNRS, IMT Toulouse France; ^7^ Université Lyon 1, Inserm Stem Cell and Brain Research Institute, INSERM U1208 Bron France; ^8^ Univ Toulouse, CNRS, CerCo Toulouse France; ^9^ School of Geography, Archaeology and Environmental Studies University of the Witwatersrand Johannesburg South Africa

**Keywords:** chimpanzee, density map, *pan* cortex, sulcal pattern, virtual endocasts

## Abstract

Human brain evolution, explored through the study of fossil endocasts, reveals key insights into changes through time of the cerebral morphology. The genus *Pan*, being the closest extant relative to humans, provides a crucial comparative basis to identify features that are unique to the human lineage, thereby enriching the understanding of adaptations that have contributed to the human evolutionary history. However, to date, there is no atlas characterising brain imprints in chimpanzee endocasts and documenting variation in their patterns. Utilising micro‐focus X‐ray Computed Tomography, 21 *Pan paniscus* and *Pan troglodytes* dry crania were scanned, and virtual endocasts were automatically reconstructed. Following the automated detection of cortical imprints and manual labelling, advanced statistical methods were applied to generate a density map that documents the distribution of sulcal imprints. Sulci that are critically discussed in palaeoneurology, such as the lunate sulcus, were reliably detected and identified on 21 endocasts, in addition to other sulci of the frontal‐, parietal‐, temporal‐, and occipital lobes, while accessory sulci such as the fronto‐marginal sulcus and pre‐lunate sulcus showed lower detection rates. This study provides the first comprehensive description of sulcal imprints on extant *Pan* endocasts and presents invaluable comparative resources for studying brain evolution from the hominin fossil record.

## Graphical abstract

This comprehensive atlas documents the frequency, position, and variability of cortical sulcal imprints across 21 extant *Pan* endocasts, providing the first quantitative baseline for comparative paleoneurology. This atlas serves as a reference for distinguishing ancestral from derived features in hominin brain evolution.

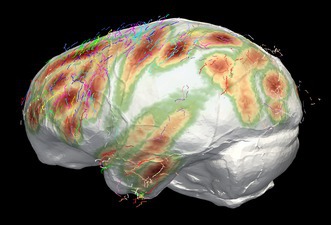

## 1 INTRODUCTION

The evolution of the hominin brain remains a central question in human evolutionary studies. Reconstructing brain evolution remains challenging because direct brain tissue does not preserve in the fossil record. Instead, palaeoneurologists rely on cranial endocasts, replicas of the internal surface of the braincase, to infer structural changes over time (Holloway et al., 2004; Neubauer, 2014). Debates about endocranial morphology began with early discoveries such as *Pithecanthropus* (Dubois, 1898) and the La Chapelle‐aux‐Saints Neanderthal (Boule & Anthony, 1911). However, the discovery of the Taung child's natural endocast in 1925 intensified discussions about when key features of the human brain emerged, particularly regarding the position of key sulci (e.g., lunate sulcus, ascending and horizontal rami) and the timing of cortical reorganisation (Dart, 1925; Falk, 1980; Holloway, 1981). Reanalyses have been challenged the interpretation of these landmark specimens for decades (e.g., Falk, 1980, 1983, 2009, 2014; Holloway, 1981, 1984; Holloway et al., 2003; Hurst et al., 2025), and the absence of consensus could be tentatively explained by the lack of reliable methods to systematically document convolutional imprints on endocasts, with observer‐dependent identifications obscuring interpretations of hominin brain evolution.

Advances in computer tomography (CT) scanning and automated image analysis have revolutionised the study of fossil hominin cranial endocasts (Beaudet et al., 2019; Zollikofer & Ponce de León, 2013). Digital endocasts can now be extracted from fossil crania non‐destructively, and computational algorithms can detect cortical sulci more objectively than traditional hand‐drawn analyses (e.g., de Jager et al., 2019). Adapting techniques from neuroimaging research, where probabilistic brain atlases are used to characterise population‐level sulcal variability (e.g., Toga & Thompson, 2001), recent studies have applied density mapping to endocasts, allowing individual sulcal imprints to be projected onto a common template to quantify positional consistency and variation across specimens (de Jager et al., 2022).

These methodological advances have been mainly applied to fossil hominins and extant humans, with limited systematic study of extant great apes. This limitation has deep implications for our understanding of the mechanisms underlying cortical evolution and distinguishing ancestral from derived features. Recent evidence indicates that brain‐specific genes did not evolve faster in humans than in *Pan* (Shi et al., 2006) and that more genes underwent positive selection in the chimpanzee lineage (Bakewell et al., 2007). Moreover, at the cellular level, chimpanzees exhibit more distinct phenotypes in prefrontal cortex interneuron distributions compared with other anthropoid primates (Sherwood et al., 2010). Indeed, ongoing debates regarding the chronology of brain changes revolve around the possibility that such changes reflect neural adaptations that are specific to the human lineage and that can contribute to the search for features that can define humans (genus *Homo*; Beaudet, 2021; Ponce de León et al., 2021; Bruner & Beaudet, 2023). For instance, recent work has identified a unique neuroanatomical feature in humans related to speech evolution: the prefrontal extent of the frontal operculum (PFOp). Located within the prefrontal cortex (i.e. anterior to the inferior precentral sulcus), the PFOp sits between the circular sulcus, which limits the insula dorsally, and the lateral surface of the Broca's area. This structure is absent in Old world monkeys, where the insula lies more posteriory (behind the precentral sulcus) and is consequently covered by the premotor operculum. However, a PFOp precursor may exist in certain individuals of the genus *Pan*, where the insula reaches the level of the inferior precentral sulcus and extends into the prefrontal territory (Amiez, Verstraete, et al., 2023). Understanding the evolutionary emergence of these features requires comparison with the ancestral condition. In *Pan*, the fronto‐orbital sulcus shows both singular versus bifurcated morphological variants (Hopkins et al., 2022). This bifurcated morphology may represent a precursor configuration before the invagination and reorganisation that created the modern human prefrontal opercular anatomy. Such features are traditionally identified by comparing hominins with genus *Pan*, which comprises chimpanzees (*Pan troglodytes*) and bonobos (*Pan paniscus*), and represents the closest living relatives of humans, with divergence from our most recent common ancestor estimated between 8 and 12 million years ago (Arnason & Janke, 2002; Langergraber et al., 2012; Moorjani et al., 2016). Accordingly, the brain of *Pan* is considered representative of the ancestral primate cortical sulcus morphology and thus lacks the derived features accumulated during hominin evolution (de Sousa & Cunha, 2012; Falk et al., 2018). However, it is essential to keep in mind that the extant population of *Pan* has had its own evolution (Wood and Lonergan, 2008).

Although foundational work by Le Gros Clark et al. (1936) and subsequent comparative descriptive studies (Connolly, 1950; Falk et al., 2018; Holloway, 1981, 1988) exist, systematic comparative descriptions of sulcal imprints on extant *Pan* endocasts using modern quantitative approaches remain limited. Recent work focusing on the brain has demonstrated significant variability in *Pan* sulcal morphology, particularly in the inferior frontal gyrus (Hopkins et al., 2022), and the superior temporal gyrus (Sathishkumar et al., 2025), suggesting that the chimpanzee/bonobo pattern, used as the ‘ancestral’ condition by palaeoneurologists (e.g., Falk, 2014; Falk et al., 2018; Holloway et al., 2004), may be more variable than previously assumed. As such, understanding which sulcal patterns, if found in hominins, are possibly inherited from our last common ancestor with *Pan* (‘ancestral’), and which can be considered as specific to the human lineage (‘derived’) is essential, particularly as new research highlights the complexity of cortical evolution in primates (Melchionna et al., 2025).

This study, therefore, aims to create the first comprehensive, modern atlas of sulcal imprints on *Pan* endocasts. By utilising automated detection algorithms and a density‐mapping approach, we generate a reproducible, high‐resolution comparative reference for studying hominin brain evolution.

## 2 MATERIALS AND METHODS

### 2.1 Sample

Twenty‐one crania (10 males and 11 females), consisting of wild‐shot adult *Pan paniscus* (bonobo; *n* = 9) and *Pan troglodytes* (chimpanzee; *n* = 12) from the Royal Museum of Central Africa, Belgium, were scanned using micro‐focus X‐ray Computed Tomography at the Centre for X‐ray Tomography of Ghent University, Belgium (Masschaele et al., 2013). The crania were scanned at a spatial resolution ranging from 0.065 to 0.085 mm^3^ (isometric).

### 2.2 Detection, identification, and analysis of sulcal imprints

**FIGURE 1:**
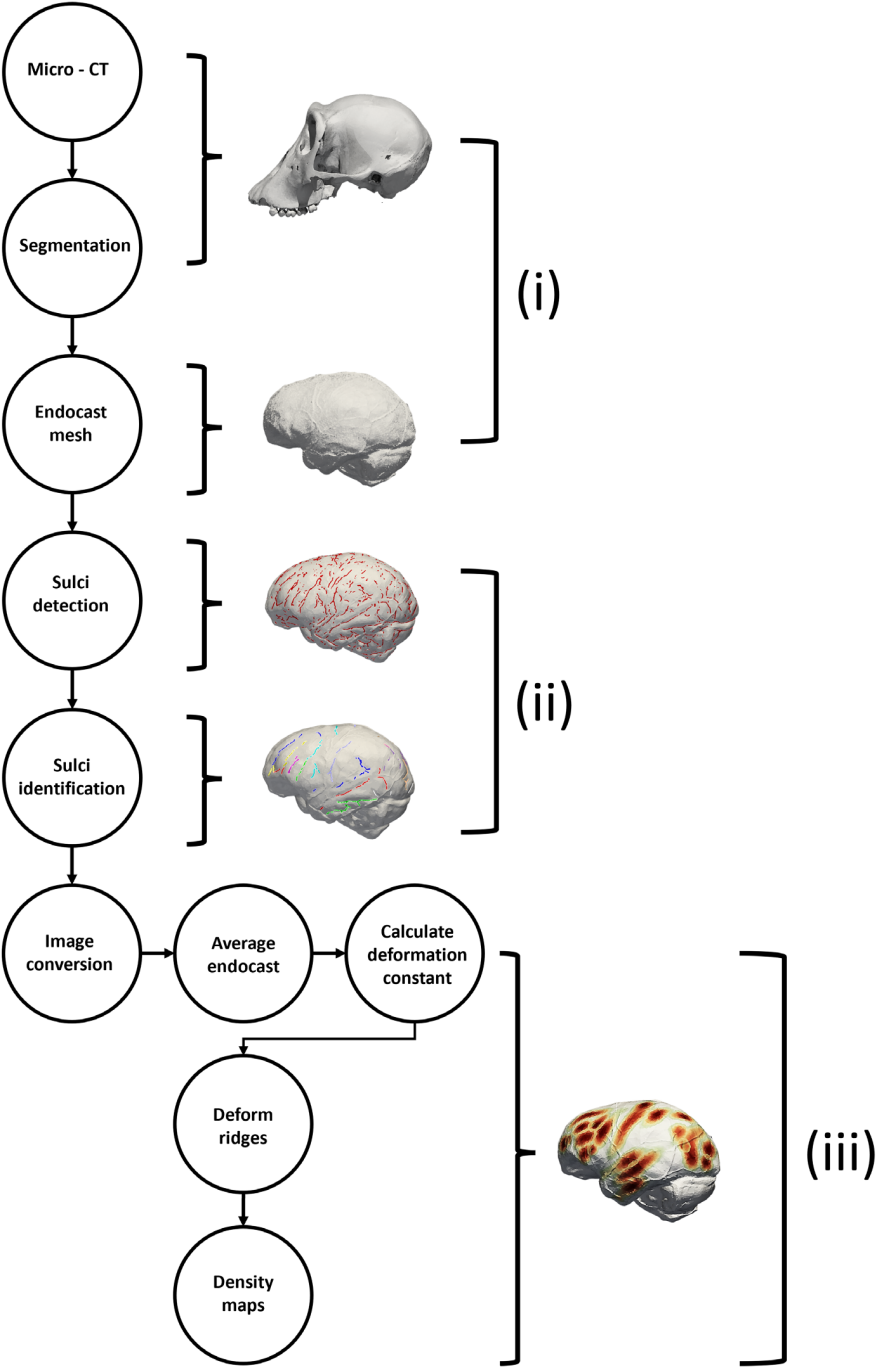
Detailed flow diagram of method used based on the protocol by de Jager et al. (2022). (i) Image acquisition and segmentation; (ii) Imprint labelling and atlas creation; (iii) Density map.

The virtual endocasts were extracted, and the cortical imprints were detected and analysed using the protocol detailed in de Jager et al. (2022). This protocol can be summarised as follows (Figure 1): (i) endocasts were automatically extracted using Endex software (Subsol et al., 2010) and Avizo v2022.2 (Thermo Fisher Inc.), and sulcal imprints were detected using an algorithm based on the concept of Yoshizawa et al. (2008); (ii) imprints on the left hemispheres (LHs) and right hemispheres (RHs) were labelled separately, and cleaned using a custom application created in MATLAB (Beaudet et al., 2016, 2019; de Jager et al., 2019), with labelling done in consultation with brain atlases and various published resources (e.g., Amiez, Sallet, et al., 2023; Connolly, 1950; Falk et al., 2018; Hopkins et al., 2022); (iii) density maps were created by projecting the sulcal imprints of the entire sample to the mean endocast created using an iterative deformation method (de Jager et al., 2022).

As an extension of this protocol, a composite variability score was computed for each sulcus to quantify spatial dispersion across individuals. For each labelled sulcus, the standard deviation of its projected coordinates along the X (medial‐lateral), Y (anterior–posterior), and Z (superior–inferior) axes on the common template was calculated, and the Euclidean norm of this 3D standard deviation vector was used as a global measure of sulcal variability. This metric, which was not included in the previous human endocast study (de Jager et al., 2022), complements the density maps by providing a quantitative assessment of both labelling consistency and biological variability. Sulci with low variability scores either reflect consistent identification across individuals or genuinely stable anatomical positions, while high variability scores may indicate greater special uncertainty in sulcal identification or true biological variation in sulcal position across *Pan* endocasts.

### 2.3 Statistical analyses

Statistical analyses were performed in R v4.3.2 (R Core Team, 2023). Associations between sulcal presence and biological variables were assessed using contingency table analysis. To test for overall differences in sulcal identification patterns between taxa (*Pan paniscus* vs. *Pan troglodytes*), sulcal frequency counts were aggregated by taxon, hemisphere, and sulcus, and Fisher's exact test was applied to the resulting frequency distribution using simulated *p*‐values with 2000 replicates where computational limits were encountered. Individual sulcus‐level comparisons between taxa were also conducted using Fisher's exact test or Chi‐square test (when cell counts were sufficient). Sex‐related differences in sulcal presence were assessed similarly. Hemispheric asymmetries in sulcal presence were evaluated using Chi‐squared or Fisher's exact test when expected frequency counts were low. *p*‐values were adjusted for multiple comparisons using the false discovery rate (FDR) method where appropriate. All tests were conducted at an alpha level of 0.05, and data visualisations were produced using the ggplot2 package (Wickham, 2016).

## 3 RESULTS

### 3.1 Description of the sulcal imprints

**FIGURE 2:**
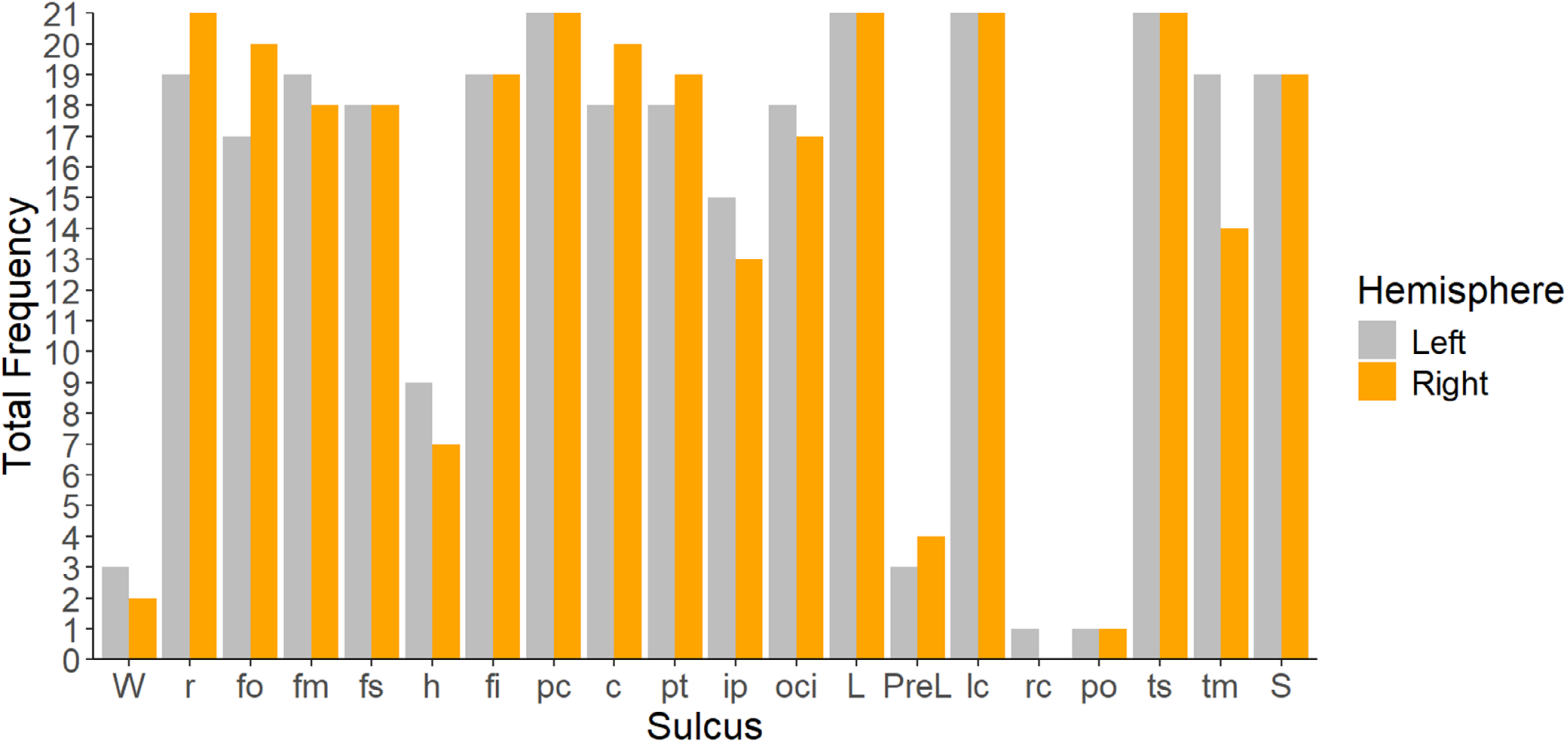
Bar chart indicating the total frequency of sulci identified in the entire *Pan* sample. c, Central sulcus; fi, Inferior frontal sulcus; fm, Middle frontal sulcus; fo, Fronto‐orbital sulcus; fs, Superior frontal sulcus; h, Horizontal ramus of inferior precentral sulcus; ip, Intraparietal sulcus; L, Lunate sulcus; lc, Lateral calcarine sulcus; oci, Inferior occipital sulcus; pc, Precentral sulcus; PreL, Pre‐lunate sulcus; pt, Postcentral sulcus; r, Sulcus rectus; rc, Retrocalcarine sulcus; S, Sylvian fissure; tm, Middle temporal sulcus; ts, Superior temporal sulcus; W, Fronto‐marginal sulcus.

In general, the sulci of the frontal, parietal, occipital, and temporal lobes were identified with high confidence across the sample. Most of the major sulcal landmarks could be reliably distinguished on both hemispheres; however, certain sulci presented more challenges in terms of identification. In particular, the fronto‐marginal sulcus of Wernicke, the horizontal ramus of the inferior precentral sulcus, and the pre‐lunate sulcus were identified in relatively few individuals (Figure 2). Of these, the pre‐lunate sulcus showed exceptionally high positional variability on the LH (28.00) but low variability on the right (7.01; Table 1), while the fronto‐marginal and horizontal ramus of the inferior precentral sulcus showed low variability when present, suggesting that their inclusion in the template reflects sparse but consistent identifications rather than widespread expression.

**TABLE 1:** Composite variability scores (%) for sulci on *Pan* endocasts.

Sulcus	Left variability	Right variability	Difference	Mean variability
Fronto‐marginal	3.12	3.81	0.69	3.46
Rectus	6.21	6.61	0.40	6.41
Fronto‐orbital	6.52	10.39	3.86	8.45
Superior frontal	13.28	13.30	0.01	13.29
Middle frontal	9.32	11.97	2.65	10.65
Horizontal ramus	7.01	4.22	2.79	5.62
Inferior frontal	7.94	7.14	0.81	7.54
Precentral	15.19	15.34	0.15	15.27
Central	18.21	16.87	1.35	17.54
Postcentral	15.66	14.87	0.79	15.27
Intraparietal	8.78	8.55	0.24	8.66
Inferior occipital	10.41	11.84	1.43	11.13
Lunate	14.61	14.74	0.13	14.67
Pre‐lunate	28.00	7.01	21.00	17.51
Lateral calcarine	9.48	8.82	0.66	9.15
Retrocalcarine	2.41			
Parieto‐occipital	1.63	2.75	1.11	2.19
Superior temporal	21.60	22.28	0.69	21.94
Middle temporal	13.86	16.13	2.27	15.00
Sylvian fissure	15.59	15.88	0.30	15.73

*Note*: Higher values indicate greater positional variability across individuals; lower values reflect more consistent sulcal expression.

#### 3.1.1 Frontal sulci

**FIGURE 3:**
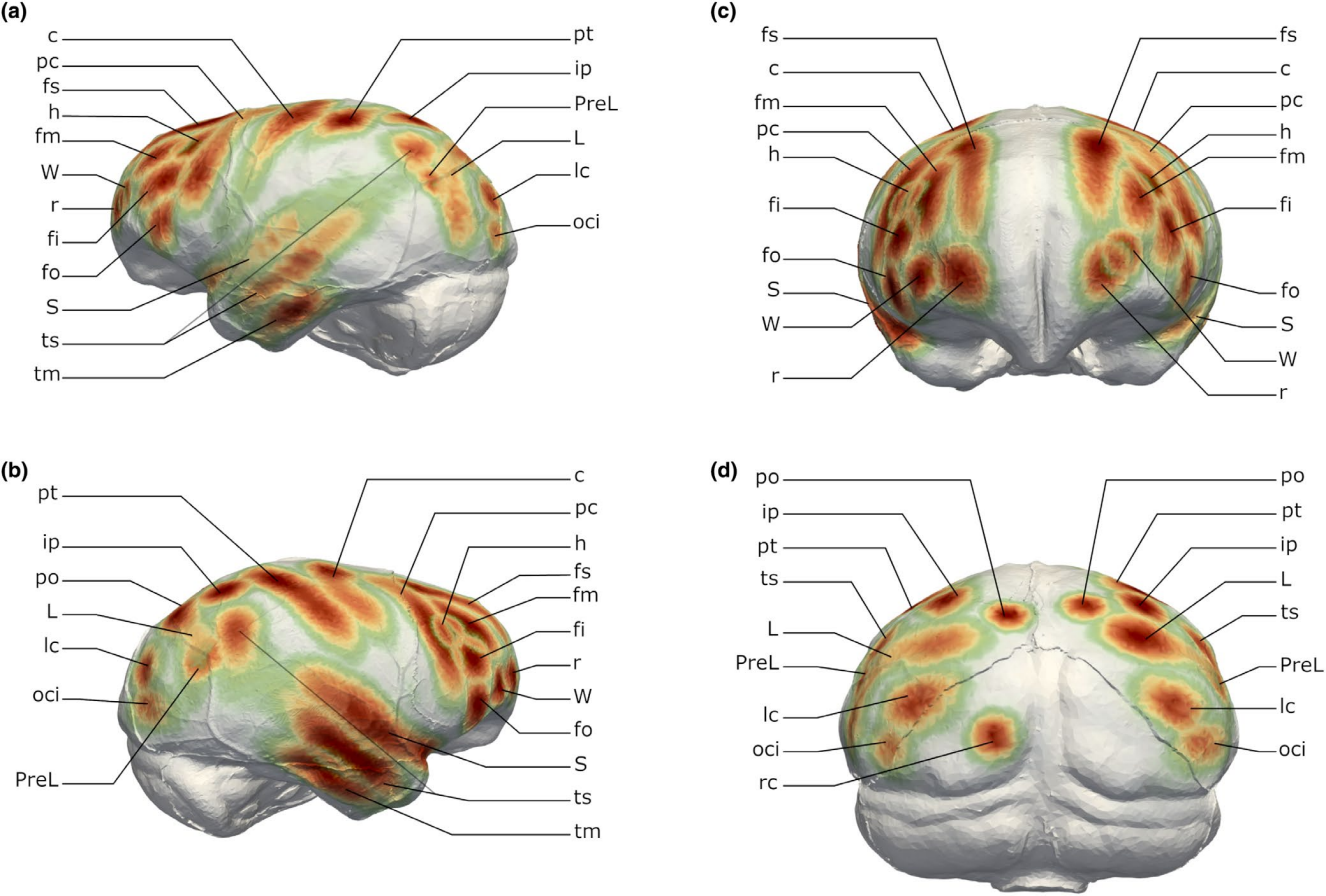
Lateral (a, b), superior (c), and posterior (d) views showing sulcal organisation and sulcal probability mapping in *Pan*. C, Central sulcus; fi, Inferior frontal sulcus; fm, Middle frontal sulcus; fo, Fronto‐orbital sulcus; fs, Superior frontal sulcus; h, Horizontal ramus of inferior precentral sulcus; ip, Intraparietal sulcus; L, Lunate sulcus; lc, Lateral calcarine sulcus; oci, Inferior occipital sulcus; pc, Precentral sulcus; po, Parieto‐occipital sulcus; PreL, Pre‐lunate sulcus; pt, Postcentral sulcus; r, Sulcus rectus; rc, Retrocalcarine sulcus; S, Sylvian fissure; tm, Middle temporal sulcus; ts, Superior temporal sulcus; W, Fronto‐marginal sulcus.

**FIGURE 4:**
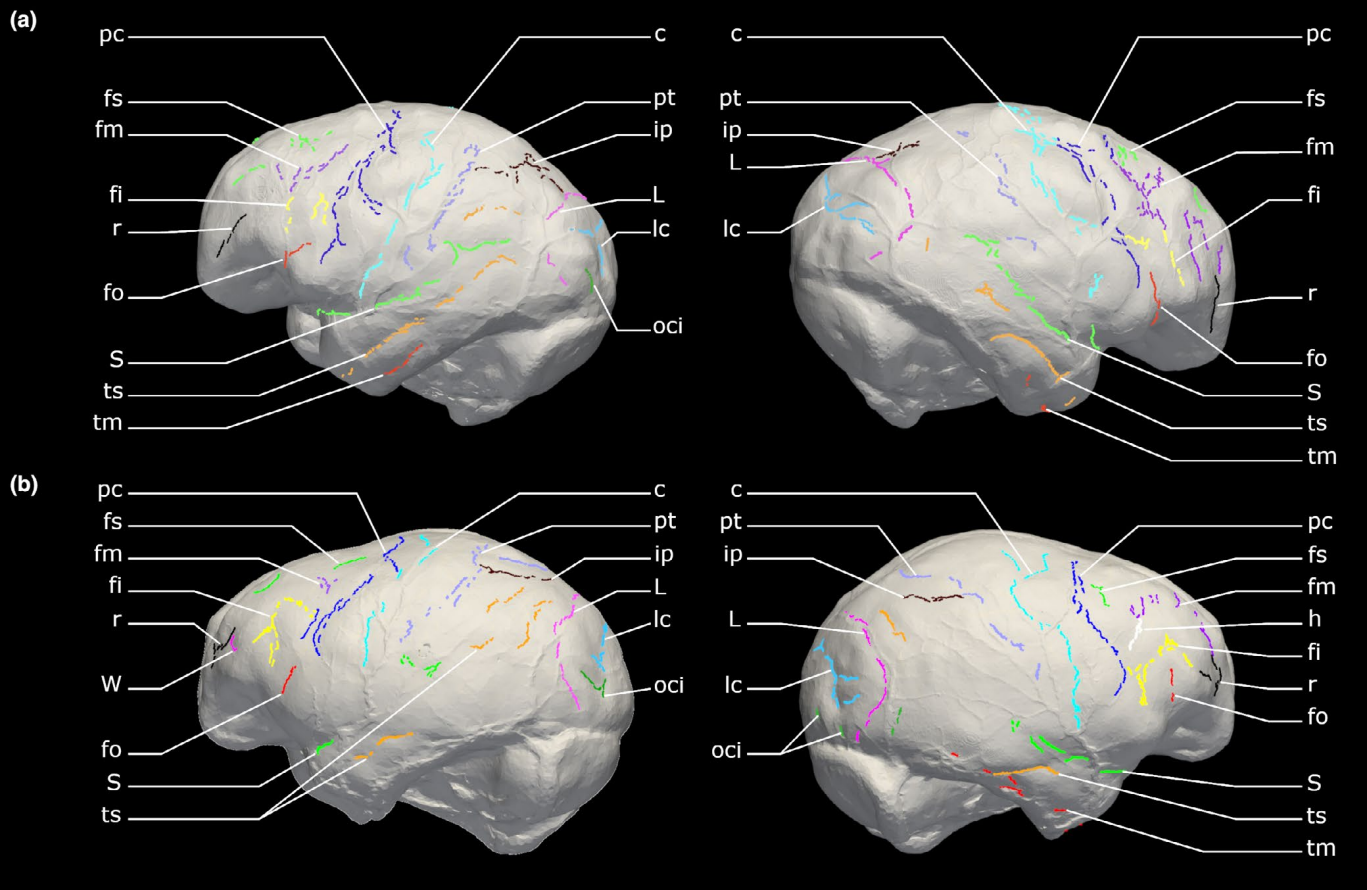
Representative examples of sulcal imprints on *Pan* endocasts in lateral left and right views. (a) *Pan paniscus*. (b) *Pan troglodytes*. C, Central sulcus; fi, Inferior frontal sulcus; fm, Middle frontal sulcus; fo, Fronto‐orbital sulcus; fs, Superior frontal sulcus; h, Horizontal ramus of inferior precentral sulcus; ip, Intraparietal sulcus; L, Lunate sulcus; lc, Lateral calcarine sulcus; oci, Inferior occipital sulcus; pc, Precentral sulcus; pt, Postcentral sulcus; r, Sulcus rectus; S, Sylvian fissure; tm, Middle temporal sulcus; ts, Superior temporal sulcus; W, Fronto‐marginal sulcus.

The sulcus rectus was reliably identified on the majority of the endocasts (LHs: 90%; RHs: 100%) as a diagonal imprint situated along the rostral margin of the frontal lobe. In most cases, this sulcus presented as a fragmented impression, although it occasionally appeared continuous with the imprints of the superior frontal sulcus and the middle frontal sulcus (Figure 4b). Variability scores for the sulcus rectus were low and symmetrical between hemispheres (LHs: 6.21; RHs: 6.61), indicating positional consistency despite its partial appearance in some specimens. In a subset of endocasts, the sulcus rectus displayed a lateral branch (LHs: 14%; RHs: 10%), which was designated as the fronto‐marginal sulcus of Wernicke (Figure 4b; Figures S1 and S2). This sulcus had very low variability (LHs: 3.12; RHs: 3.81), which confirmed consistent anatomical expression across hemispheres. The density map produced a continuous projection (Figure 3). The middle frontal sulcus occupied the middle frontal convolution (LHs: 90%; RHs: 86%; Figure 2) and was similarly consistent across hemispheres. On the inferior frontal convolution, the most prominent sulcal impression was that of the fronto‐orbital sulcus (LHs: 81%; RHs: 95%), which, in the majority of cases, appeared almost continuous with the imprints of both the inferior precentral sulcus and the inferior frontal sulcus (Figure 4a; Figures S1 and S2). Notably, the fronto‐orbital sulcus had markedly higher variability on the right hemisphere (LHs:6.52; RHs: 10.39), suggesting greater positional uncertainty or population‐level variation on that side. The horizontal ramus of the inferior precentral sulcus could be identified in fewer than half of the endocasts (LHs: 43%; RHs: 33%); however, the identification of this sulcus remains uncertain, and some cases may represent misidentifications (Table 1). The precentral sulcus was clearly observed in all specimens (100%) as a distinct diagonal imprint crossing the coronal suture in most samples (see Figure 3). Most notably, the inferior portion of the precentral sulcus consistently appeared anterior to the coronal suture, while more distorted or ambiguous patterns were observed towards the sagittal margin of the hemisphere (Figures S1 and S2).

#### 3.1.2 Temporal sulci

The Sylvian fissure (or lateral sulcus) was most frequently observed (LHs: 90%; RHs: 90%), particularly towards the rostral portion of the temporal lobe. In instances where the Sylvian fissure could not be confidently identified, the impression appeared to be obscured or distorted, often by the imprint the middle meningeal artery. Despite these interruptions, the fissure showed moderate spatial consistency across specimens (LHs: 6.10; RHs: 6.74). Directly inferior to the Sylvian fissure, the superior temporal sulcus was consistently identified across all endocasts and presented with relatively low positional variability (LHs: 5.43; RHs: 5.71), suggesting a stable and well‐defined expression in both hemispheres, confirmed by a broad, high‐density groove running along the lateral temporal lobe (Figure 3). The middle temporal sulcus (LHs: 90%; RHs: 67%) was typically observed immediately inferior to the superior temporal sulcus, most commonly extending towards the temporal pole (Figure 4l; Figures S1 and S2). However, it exhibited a noticeable hemispheric asymmetry in positional variability (LHs: 5.96; RHs: 8.87), indicating greater spatial dispersion in the right hemisphere.

#### 3.1.3 Parietal sulci

Marking the boundary between the frontal and the parietal lobes, the central sulcus (LHs: 86%; RHs: 95%; Figure 4; Figures S1 and S2) was identified as a distinct impression situated directly posterior to the coronal suture, which appears as a sharp high‐density cluster in the population‐averaged map (Figure 3). It was among the most consistently identified sulci, and Composite Variability scores confirmed this high spatial reliability (LHs: 4.20; RHs: 4.94). On the parietal lobe, the postcentral sulcus (LHs: 86%; RHs: 90%) was identified in the majority of specimens. It typically formed a crescent‐shaped impression extending from the caudal extremities of the Sylvian fissure and appeared, in many cases, to continue seamlessly into the imprint of the intraparietal sulcus (LHs: 71%; RHs: 62%), which is evident from the density maps (Figure 3). Variability scores for the postcentral sulcus were moderate and symmetrical (LHs: 6.21; RHs: 6.68), while the intraparietal sulcus showed slightly greater positional variability on the right (LHs: 7.19; RHs: 8.63). The inferior parietal lobule was commonly occupied by the imprints of the posterior rami of the superior temporal sulcus, which delineated the boundaries of the angular marginal gyri (Figure 4).

#### 3.1.4 Occipital sulci

The occipital lobe was bordered caudally by the distinct imprint of the lunate sulcus, which was identified on all the endocasts examined as a prominent crescent‐shaped imprint (see Figure 3). This sulcus showed very low positional variability across hemispheres (LHs: 3.94; RHs: 4.26), indicating strong spatial conservation. Immediately caudal to the lunate sulcus, the lateral calcarine sulcus was consistently identified in both hemispheres (LHs: 100%; RHs: 100%; Figure 4; Figures S1 and S2) and presented the lowest variability scores in the occipital region (LHs: 86%; RHs 81%), being positioned laterally on the occipital cortex, with relatively symmetrical variability (LHs: 6.38; RHs: 6.50). In a subset of hemispheres, a clear imprint of the pre‐lunate sulcus was identified (RHs: 14%; RHs: 19%), showing higher variability (LHs: 9.37; RHs: 9.93), consistent with its infrequent and variable expression. In a single isolated case, the retrocalcarine sulcus was also detected (LHs: 5%; Figure 2), but no variability score could be calculated.

### 3.2 Density maps

The density maps represent the spatial distribution and overlap of sulcal imprints across the 21 *Pan* endocasts (Figure 3). High‐density areas (indicated in red) represent regions where sulcal imprints were consistently identified across multiple specimens, while low‐density areas (green) indicate lower detection frequencies.

The imprints of the sulcus rectus and the fronto‐marginal sulcus show dense clusters along the frontal margin on both hemispheres, with some overlapping distributions observed on the LH (Figure 3c). Lateral to these, on the lateral margin of the frontal lobe, the density distribution of the fronto‐orbital sulcus could be observed showing positional overlap with the inferior precentral and inferior frontal sulci (Figure 3a,b). Along the medial margin of each hemisphere, the distribution of the superior frontal sulcus can be observed, showing higher density at its posterior origins, with some overlap with the cluster of the middle frontal sulcus on the right hemisphere (Figure 3c).

Occupying the middle of the frontal lobe, overlapping clusters of the middle frontal sulcus, the horizontal ramus of the inferior precentral sulcus, and the inferior frontal sulcus can be observed (Figure 3a,b). Interestingly, on both hemispheres, the continuity between the middle frontal sulcus and the sulcus rectus could be observed, as well as overlap with the horizontal ramus of the inferior precentral sulcus at the posterior end of the middle frontal sulcus. On the right hemisphere, major overlap can be observed between the inferior precentral sulcus and its surrounding middle frontal and fronto‐orbital sulci (Figure 3b). The precentral sulcus showed its highest density of identification on the inferior lateral frontal lobe on both hemispheres, with its density distribution showing it clearly crossing the imprint of the coronal suture towards the medial margin of the hemisphere (Figure 3a,b). Clear overlap could be observed between the clusters of the inferior precentral sulcus, the fronto‐orbital sulcus, and the inferior frontal sulcus.

Separating the frontal lobe and the parietal lobe, the density distribution of the central sulcus can be observed showing its highest point of identification closer to the medial margin of both hemispheres (Figure 3a,b). The postcentral sulcus can be observed at its highest density in the middle of the parietal lobe on the left and RHs; however, it shows a larger distribution on the right hemisphere spanning the entirety of the parietal lobe (Figure 3b). Clearly overlapping with the postcentral sulcus, the distribution of the intraparietal sulcus can be observed spreading diagonally over the posterior parietal lobe and overlapping with the density map of the lunate sulcus at its posterior extremity (Figure 3c). The lunate sulcus can be observed as a crescent‐shaped distribution on the posterior parietal lobe on both hemispheres. Although it is more complete on the LH, a more dense distribution can be observed superiorly on the parietal lobe on the right hemisphere (Figure 3d). On both hemispheres, directly medial to the lunate sulcus, the density distribution of the parieto‐occipital sulcus can be observed. On both hemispheres, a small distribution can be observed of the pre‐lunate sulcus, with major overlap between the lunate sulcus and the posterior rami of the superior temporal sulcus. Posterior to the lunate sulcus, two clusters can be observed, both anterior to the lambdoid suture: these are the clusters of the lateral calcarine sulcus medially and the inferior occipital sulcus laterally. On the LH, close to the medial margin of the occipital lobe, a clear distribution of the lateral calcarine sulcus can be observed.

Laterally, separating the temporal lobe from the parietal lobe, the distribution of the Sylvian fissure can be observed, interestingly showing higher density on the right hemisphere. Inferior to it, the superior temporal sulcus shows higher densities on the right hemisphere, and on both hemispheres, a high‐density distribution of the superior temporal sulcus can be observed directly anterior to the lunate sulcus. Inferior on the temporal margin, the distribution of the middle temporal sulcus can be observed and, similar to the superior temporal sulcus, shows higher densities on the right hemisphere.

### 3.3 Statistical analysis

Comparative analyses revealed a significant association between taxon and overall distribution of sulcal identification frequencies (Fisher's exact test, *p* < 0.001), with *Pan troglodytes* showing higher sulcal identification rates than *Pan paniscus* across the dataset. However, when individual sulci were tested separately for taxonomic differences, no sulcus showed significant differences after correction for multiple comparisons (all FDR‐adjusted *p* > 0.05), suggesting that the overall pattern reflects general differences in preservation or identification rather than specific anatomical distinctions.

Similarly, biological sex showed a significant association with sulcal variability patterns across both hemispheres and both *Pan* species (Fisher's exact test, *p* < 0.001), although individual sulcus‐level comparisons revealed no consistent sex‐specific differences after multiple comparison correction.

No significant hemispheric asymmetry in sulcal presence was detected at the population level (*p* > 0.05), indicating relatively symmetric sulcal preservation across LHs and RHs.

## 4 DISCUSSION

This study provides a comprehensive description of sulcal imprints on *Pan* endocasts using high‐resolution imaging and quantitative analytical methods. By combining automated imprint detection with density mapping and composite variability scoring (de Jager et al., 2019, 2022), we generated visualisations and metrics that characterise sulcal organisation in *Pan* with greater precision and reproducibility than previous approaches. This comparative framework is essential for interpreting sulcal patterns in fossil hominin endocasts, in order to distinguish ‘ancestral’ and ‘derived’ features associated with hominin brain evolution.

### 4.1 Key sulci in a palaeoneurological context

Most primary sulcal landmarks could be reliably distinguished on both hemispheres, though certain sulci presented identification challenges. The fronto‐marginal sulcus, horizontal ramus of the inferior precentral sulcus, and the pre‐lunate sulcus were poorly preserved and infrequently detected across the sample. However, when identified, they often showed relatively low variability scores, suggesting that detection frequency rather than positional inconsistency limits their utility as a comparative landmark. The most spatially stable sulci with high detection rates included the lunate sulcus and the central sulcus, indicating these structures can be reliably identified at consistent anatomical positions across individuals. These quantitative assessments address long‐standing challenges in palaeoneurology related to subjective interpretation and limited reproducibility (Holloway, 2018; Neubauer, 2014), providing more rigorous foundations for fossil comparisons.

The lunate sulcus has been a subject of enduring interest in palaeoneurology due to historical disputes (Dart, 1925; Falk, 1980; Holloway, 1981). In this study, the lunate sulcus was reliably identified as a distinct crescent‐shaped imprint anterior to the imprint of the lambdoid suture in all specimens examined, with very low positional variability across hemispheres. Starting inferiorly in close proximity to the origin of the sigmoid sinus and terminating superiorly directly anterior to the position of lambda, the lunate sulcus represents one of the most consistent landmarks on *Pan* endocasts. Interestingly, the highest probability of identifying the lunate sulcus of the LH occurs towards the inferior border, while on the right hemisphere, it is the highest on the superior border, potentially reflecting directional asymmetries previously reported in chimpanzee endocrania (Neubauer et al., 2020). This baseline of sulcal expression and positional consistency in extant *Pan* is critical for assessing claims of derived lunate sulcus positions in fossil hominins.

The fronto‐orbital sulcus is of particular evolutionary significance due to its relationship to language areas and its absence in extant human brains. This sulcus is a common feature in *Australopithecus* and *Paranthropus* (Falk, 2014), as well as early *Homo*, with reported derived reorganisation appearing in *Homo* around 1.7 to 1.5 million years ago (Ponce de León et al., 2021). In our sample, the fronto‐orbital sulcus showed high‐identification rates, and its imprint frequently appeared continuous with the inferior precentral sulci on the dorsal surface of the inferior frontal lobule, correlating with observations by Le Gros Clark et al. (1936). However, it exhibited greater positional variability than most stable landmarks, particularly on the right hemisphere. This pattern is consistent with documented intraspecific variation in fronto‐orbital morphology in *Pan*, including singular and bifurcated configurations observed in MRI studies (Hopkins et al., 2022). Unfortunately, these morphological variants could not be distinguished from this sample, highlighting a possible limitation of endocast‐based analysis: while cross‐topographical features are preserved, finer anatomical details that may be functionally significant are often obscured.

The intraparietal sulcus plays an important role in identifying the position of the lunate sulcus in both extant *Pan* and early hominin endocasts, as it is known to terminate posteriorly against the lunate sulcus in its ancestral form, a relationship that changes in the derived human configuration (Connolly, 1950). The density maps generated in this study clearly demonstrate this termination pattern, with the intraparietal sulcus showing seamless continuation with the lunate sulcus in approximately 45% of hemispheres (Figures S1 and S2). This provides an important comparative baseline: fossil specimens showing this ancestral intraparietal–lunate relationship can be confidently distinguished from those showing derived configurations where these sulci are separated by expanded parietal cortex.

### 4.2 Interspecific and intraspecific variation

Statistical analysis revealed significant associations between taxon and sulcal imprint visibility, as well as between biological sex and sulcal observations. While no individual sulcus showed significant taxonomic differences after correction for multiple comparisons, *Pan troglodytes* demonstrated higher overall sulcal identification rates than *Pan paniscus*, suggesting that taxonomic differences and sexual dimorphic variation influence sulcal expression on endocasts through general differences in preservation quality or cranial morphology rather than specific neuroanatomical distinctions. However, no significant hemispheric asymmetry in sulcal presence was detected. Recent work has documented considerable variability in *Pan* sulcal morphology, particularly in the inferior frontal gyrus (Hopkins et al., 2022; Nolan et al., 2026) and superior temporal sulcus (Hopkins et al., 2023; Sathishkumar et al., 2025). These findings suggest that the ‘ancestral’ condition represented by extant *Pan* may be more polymorphic than previously assumed, with implications for how we interpret the fossil record. The presence of interspecific and intraspecific variation in extant taxa complicates straightforward ‘ancestral‐derived’ interpretations, as features once considered diagnostic of derived status may fall within the range of variation present in ancestral variations.

### 4.3 Limitations and future directions

Several limitations should be acknowledged. Endocasts capture only the external brain surface and are influenced by meninges, vascular structures, and cranial sutures, meaning sulcal imprints represent partial reflections of brain morphology (Neubauer, 2014). Numerous anatomical structures contribute to the distortion of sulcal imprints, causing missing information, particularly in regions where the brain‐endocast relations are poor (Balzeau et al., 2026; Dumoncel et al., 2021). The automated detection protocol reduces but does not eliminate subjective judgement in sulcal labelling, particularly in poorly preserved regions. Sample size, while larger than previous *Pan* endocast studies, remains too small to reliably detect species‐level inference, limiting our ability to detect subtle taxonomic or sex‐related differences. Future studies with expanded samples could refine these comparative datasets and potentially incorporate deep learning approaches for automated sulcal prediction and identification, though challenges related to incomplete preservation and missing data would need to be addressed.

Despite these constraints, this study provides the first modern comprehensive atlas of sulcal imprints on *Pan* endocasts. Using automated techniques to detect and describe sulcal imprints on *Pan* endocasts in combination with descriptions of extant human endocasts provides invaluable comparative resources for studying brain evolution in the fossil record (de Jager et al., 2019, 2022). The density maps, variability metrics, and statistical analyses offer a robust comparative framework for interpreting fossil hominin endocasts and contribute to ongoing debates about the tempo and mode of cortical reorganisation in human evolutionary history.

## AUTHOR CONTRIBUTIONS

Designed/performed research: E.d.J., A.B.; contributed new reagents/analytical tools: E.d.J., L.R., M.M., collected samples: E.d.J., A.B.; analysed/interpreted data: E.d.J., E.G., C.A., C.F., M.M., A.B.; wrote/revised the paper: E.d.J., E.G., C.A., C.F., M.M., A.B.

## Supporting information


**Figure S1.** Diagram of 12 *Pan* endocasts in lateral left and right views. (A–G) Pan paniscus. (H–L) Pan troglodytes. Abbreviations: c: central sulcus; fi: inferior frontal sulcus; fm: middle frontal sulcus; fo: fronto‐orbital sulcus; fs: superior frontal sulcus; h: horizontal ramus of inferior precentral sulcus; ip: intraparietal sulcus; L: lunate sulcus; lc: lateral calcarine sulcus; oci: inferior occipital sulcus; pc: precentral sulcus; PreL: pre‐lunate sulcus; pt: postcentral sulcus; r: sulcus rectus; rc: retrocalcarine sulcus; S: Sylvian fissure; tm: middle temporal sulcus; ts: superior temporal sulcus; W: fronto‐marginal sulcus.
**Figure S2.** Diagram of nine *Pan* endocasts in lateral left and right views. (A–C) Pan paniscus. (D–I) Pan troglodytes. Abbreviations: c: central sulcus; fi: inferior frontal sulcus; fm: middle frontal sulcus; fo: fronto‐orbital sulcus; fs: superior frontal sulcus; h: horizontal ramus of inferior precentral sulcus; ip: intraparietal sulcus; L: lunate sulcus; lc: lateral calcarine sulcus; oci: inferior occipital sulcus; pc: precentral sulcus; PreL: pre‐lunate sulcus; pt: postcentral sulcus; r: sulcus rectus; rc: retrocalcarine sulcus; S: Sylvian fissure; tm: middle temporal sulcus; ts: superior temporal sulcus; W: fronto‐marginal sulcus.

## Data Availability

The data that support the findings of this study are available on request from the corresponding author. The data are not publicly available due to privacy or ethical restrictions.
