# Supplementary material for: Atlas of cortical sulcal imprints on Pan endocasts
Source: J Anat. 2026 Jun 7;249(6):1357–67. doi: 10.1111/joa.70185 (PMC13398787; doi:10.1111/joa.70185)
Supplement: Supplementary file 1 [file JOA-249-1357-s001.zip › joa70185-sup-0002-FigureS1-S2@SI_de Jager et al.docx]

**Supplementary Figure 1:** Diagram of twelve Pan endocasts in lateral left and right views. **A-G:** Pan paniscus. **H-L:** Pan troglodytes. Abbreviations: **c:** central sulcus; **fi:** inferior frontal sulcus; **fm:** middle frontal sulcus; **fo:** fronto-orbital sulcus; **fs:** superior frontal sulcus; **h:** horizontal ramus of inferior precentral sulcus; **ip:** intraparietal sulcus; **L:** lunate sulcus; **lc:** lateral calcarine sulcus; **oci:** inferior occipital sulcus; **pc:** precentral sulcus; **PreL:** prelunate sulcus; **pt:** postcentral sulcus; **r:** sulcus rectus; **rc:** retrocalcarine sulcus; **S:** Sylvian fissure; **tm:** middle temporal sulcus; **ts:** superior temporal sulcus; **W:** fronto-marginal sulcus.

**Supplementary Figure 2:** Diagram of nine Pan endocasts in lateral left and right views. **A-C:** Pan paniscus. **D-I:** Pan troglodytes. Abbreviations: **c:** central sulcus; **fi:** inferior frontal sulcus; **fm:** middle frontal sulcus; **fo:** fronto-orbital sulcus; **fs:** superior frontal sulcus; **h:** horizontal ramus of inferior precentral sulcus; **ip:** intraparietal sulcus; **L:** lunate sulcus; **lc:** lateral calcarine sulcus; **oci:** inferior occipital sulcus; **pc:** precentral sulcus; **PreL:** prelunate sulcus; **pt:** postcentral sulcus; **r:** sulcus rectus; **rc:** retrocalcarine sulcus; **S:** Sylvian fissure; **tm:** middle temporal sulcus; **ts:** superior temporal sulcus; **W:** fronto-marginal sulcus.
