# Supplementary figures and images for: Atlas of cortical sulcal imprints on Pan endocasts
Source: J Anat. 2026 Jun 7:10.1111/joa.70185. Online ahead of print. doi: 10.1111/joa.70185 (PMC13398787; doi:10.1111/joa.70185)

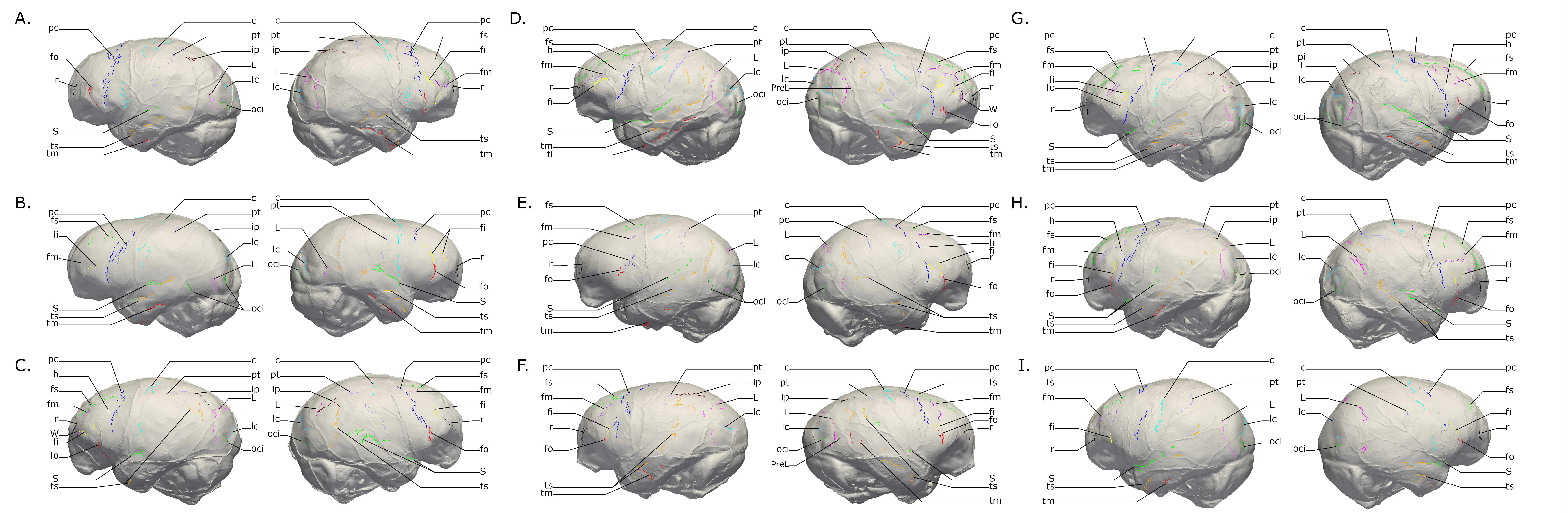

Supplement: Supplementary file 1 — Figure S1. Diagram of 12 Pan endocasts in lateral left and right views. (A–G) Pan paniscus. (H–L) Pan troglodytes. Abbreviations: c: central sulcus; fi: inferior frontal sulcus; fm: middle frontal sulcus; fo: fronto‐orbital sulcus; fs: superior frontal sulcus; h: horizontal ramus of inferior precentral sulcus; ip: intraparietal sulcus; L: lunate sulcus; lc: lateral calcarine sulcus; oci: inferior occipital sulcus; pc: precentral sulcus; PreL: pre‐lunate sulcus; pt: postcentral sulcus; r: sulcus rectus; rc: retrocalcarine sulcus; S: Sylvian fissure; tm: middle temporal sulcus; ts: superior temporal sulcus; W: fronto‐marginal sulcus. Figure S2. Diagram of nine Pan endocasts in lateral left and right views. (A–C) Pan paniscus. (D–I) Pan troglodytes. Abbreviations: c: central sulcus; fi: inferior frontal sulcus; fm: middle frontal sulcus; fo: fronto‐orbital sulcus; fs: superior frontal sulcus; h: horizontal ramus of inferior precentral sulcus; ip: intraparietal sulcus; L: lunate sulcus; lc: lateral calcarine sulcus; oci: inferior occipital sulcus; pc: precentral sulcus; PreL: pre‐lunate sulcus; pt: postcentral sulcus; r: sulcus rectus; rc: retrocalcarine sulcus; S: Sylvian fissure; tm: middle temporal sulcus; ts: superior temporal sulcus; W: fronto‐marginal sulcus. [file JOA-9999-0-s001.zip › joa70185-sup-0003-FigureS2@Figure S2.png]

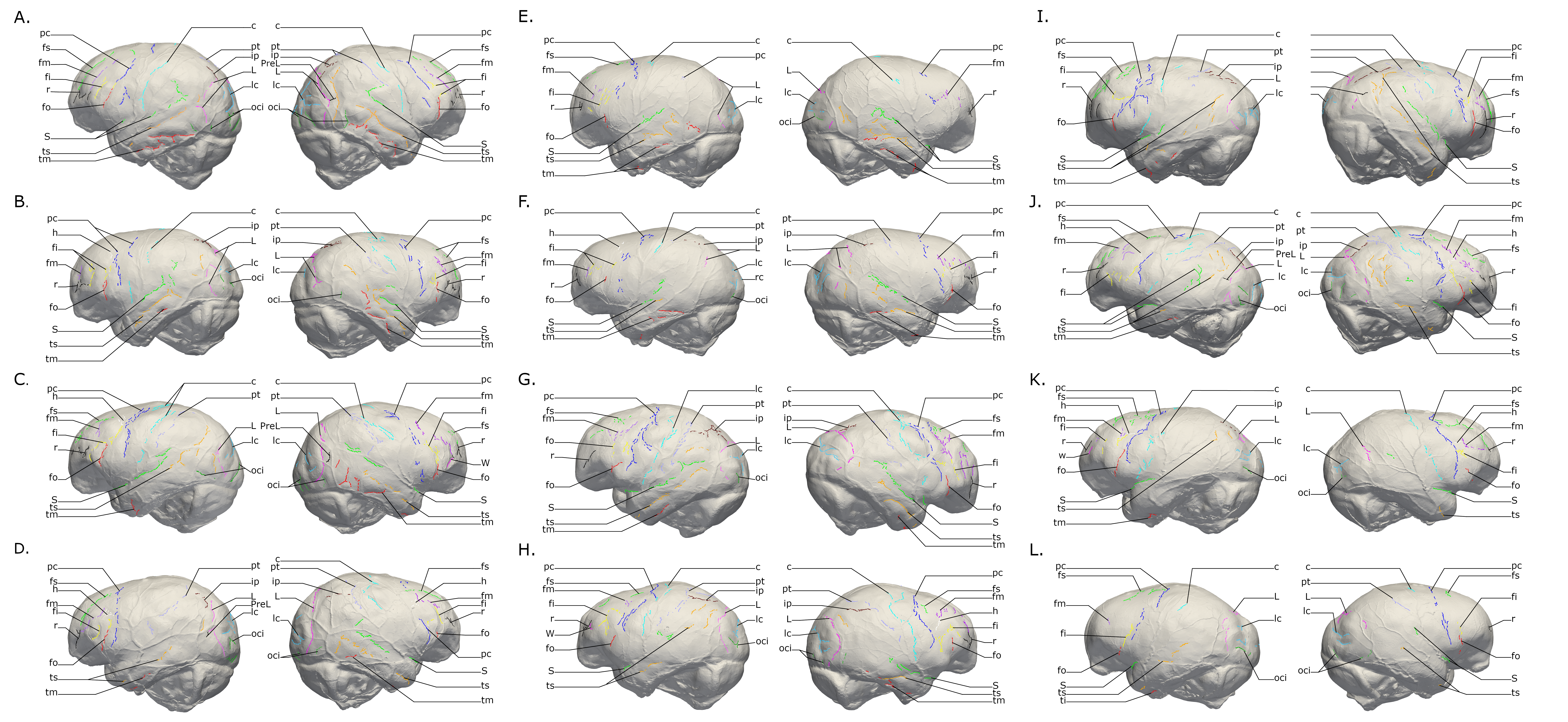

Supplement: Supplementary file 1 — Figure S1. Diagram of 12 Pan endocasts in lateral left and right views. (A–G) Pan paniscus. (H–L) Pan troglodytes. Abbreviations: c: central sulcus; fi: inferior frontal sulcus; fm: middle frontal sulcus; fo: fronto‐orbital sulcus; fs: superior frontal sulcus; h: horizontal ramus of inferior precentral sulcus; ip: intraparietal sulcus; L: lunate sulcus; lc: lateral calcarine sulcus; oci: inferior occipital sulcus; pc: precentral sulcus; PreL: pre‐lunate sulcus; pt: postcentral sulcus; r: sulcus rectus; rc: retrocalcarine sulcus; S: Sylvian fissure; tm: middle temporal sulcus; ts: superior temporal sulcus; W: fronto‐marginal sulcus. Figure S2. Diagram of nine Pan endocasts in lateral left and right views. (A–C) Pan paniscus. (D–I) Pan troglodytes. Abbreviations: c: central sulcus; fi: inferior frontal sulcus; fm: middle frontal sulcus; fo: fronto‐orbital sulcus; fs: superior frontal sulcus; h: horizontal ramus of inferior precentral sulcus; ip: intraparietal sulcus; L: lunate sulcus; lc: lateral calcarine sulcus; oci: inferior occipital sulcus; pc: precentral sulcus; PreL: pre‐lunate sulcus; pt: postcentral sulcus; r: sulcus rectus; rc: retrocalcarine sulcus; S: Sylvian fissure; tm: middle temporal sulcus; ts: superior temporal sulcus; W: fronto‐marginal sulcus. [file JOA-9999-0-s001.zip › joa70185-sup-0001-FigureS1@Figure S1.png]
